# Elastic Stable Intramedullary Nailing for Treatment of Pediatric Tibial Fractures: A 20-Year Single Center Experience of 132 Cases

**DOI:** 10.3390/children9060845

**Published:** 2022-06-07

**Authors:** Zenon Pogorelić, Viktor Vegan, Miro Jukić, Carlos Martin Llorente Muñoz, Dubravko Furlan

**Affiliations:** 1 Department of Pediatric Surgery, University Hospital of Split, 21000 Split, Croatia; mirojukic.mefst@gmail.com (M.J.); dfurlan@inet.hr (D.F.); 2 Department of Surgery, School of Medicine, University of Split, 21000 Split, Croatia; vv11398@gmail.com; 3 Surgical Clinic Medix-Muñoz, 28 000 Madrid, Spain; llorentecm@gmail.com

**Keywords:** titanium elastic nails, tibial fracture, children, elastic stable intramedullary nailing (ESIN)

## Abstract

Objective: The aim of this study was to analyze the outcomes and complications in children treated with elastic stable intramedullary nailing (ESIN) for tibial fractures. Methods: The study included 132 patients (92 males) with a median age of 11 years (IQR 10, 15) treated with ESIN for displaced tibial shaft fractures or dia-metaphyseal distal tibial fractures from March 2002 to March 2022. The median follow-up was 118.5 months (IQR 74.5, 170). The primary outcome was success rate, while secondary outcomes were the time of bone healing, length of hospital stay, and associated injuries. Demographic data, type and nature of fracture, indication for surgery, healing time, operative time, complications of treatment, and time to implant removal were recorded. Results: Complete radiographic healing was achieved at a median of 7 weeks (IQR 6, 9). Most of the patients (*n* = 111; 84.1%) had fractures localized in the shaft of the tibia. The most common injuries were acquired by road traffic accidents (*n* = 42) and by a fall in the same level (*n* = 29), followed by injuries from sport activities (*n* = 21) or motorbike accidents (*n* = 18). Associated injuries were reported in 37 (28%) children. Fractures were closed in the majority of the children (*n* = 100; 76%), while 32 (24%) children presented with an open fracture. Children with open fractures were significantly older than children with closed fractures (13.5 years (IQR 10, 15) vs. 11 years (IQR 8.5, 14.5); *p* = 0.031). Furthermore, children with open fractures had a significantly longer hospital stay (7 days (IQR 5, 9) vs. 3 days (IQR 3, 6); *p* = 0.001), a higher rate of associated injuries (*n* = 14 (43.7%) vs. *n* = 23 (23%); *p* = 0.022), and a higher rate of postoperative complications (*n* = 7 (21.9%) vs. *n* = 8 (8%); *p* = 0.031). No intraoperative complications were recorded. A total of 15 (11.4%) postoperative complications were recorded. Most complications (60%) were minor complications, mostly related to the wound at the nail insertion site and were managed conservatively. A total of six (4.5%) patients required reoperation due to angulation of the fragments (*n* = 5) or refracture (*n* = 1). Conclusion: ESIN is a minimally invasive bone surgery technique and is a highly effective treatment for pediatric tibial unstable fractures with a low rate of complications. Based on the given results, surgical stabilization of the tibial fractures using titanium intramedullary nailing can be safely performed without casting with early physiotherapy.

## 1. Introduction

Lower leg fractures in children are the third most common location after forearm and femur fractures [1,2]. Among all the lower leg fractures, isolated tibial fractures are the most frequent and account for about 70% of the fractures. Both bones are broken in 30% of patients, and isolated fibular fractures are rare [3]. According to the type, the transverse or short oblique fractures of the tibia are most commonly seen [4,5]. The tibia is most susceptible to trauma in the area of transition from the middle to the distal third due to anatomical changes in the cross-section of the bone from triangular to round in shape [6]. The choice of the treatment method depends on the bone age, body weight and general condition of the patient, and the type, angle, and the location of the fracture. In the earliest and preschool age, fractures are usually treated conservatively because short-term immobilization is sufficient to stabilize fractures with periosteal callus and the high potential for remodeling corrects almost all deformities. In older children, bone healing is slower, and the required stability of the fragments over time is difficult to achieve with immobilization. In total, only 10% of all fractures in children require surgical treatment [7]. Several recent studies pointed out that pediatric tibial fractures have been increasingly treated by a surgical approach [8,9]. According to good medical practice and guidelines, surgery should be undertaken only in cases of severe dislocation that could not be reduced, fracture instability, open fractures, compartment syndrome, neurovascular dysfunction, failed non-operative management, or in cases of polytrauma [10]. Surgical techniques for the treatment of tibial fractures in children include elastic stable intramedullary osteosynthesis with titanium nails (ESIN), Kirschner wires, osteosynthetic plate placement, and external fixation [11,12]. The use of elastic stable intramedullary nails has almost become a routine treatment method for pediatric diaphyseal fractures of long bones in the last few years. There is much evidence that proves this method has the benefits of early immediate stability to the involved bone segment, which permits early mobilization and return to normal activities of the patients without immobilization, with very low complication rates [13,14,15,16]. The benefits of ESIN, compared to other surgical techniques, include shorter surgical time, minimal soft-tissue dissection, improved cosmesis, less pain, early mobilization, and relatively easy implant removal [13,14,15,16]. Although many authors recommend immobilization in the postoperative period, our previous study clearly showed that immobilization is not necessary and that there is no increased number of complications if immobilization did not occur [13,15,17,18,19]. The aim of this retrospective analysis was to evaluate the outcomes of treatment and complication rates of tibial fractures treated with ESIN in children and adolescents in a representative cohort of 132 patients in order to underline the safeness and efficiency of this technique. Moreover, our goal was to present that immobilization is not required after ESIN osteosynthesis.

## 2. Materials and Methods

### 2.1. Patients

A retrospective search of 132 children (92 males) who underwent ESIN for tibial fractures, from March 2002 to March 2022 in a Clinic of Pediatric Surgery at University Hospital of Split, Croatia, was performed. The median follow-up was 118.5 (IQR 74.5, 170) months, while the median age was 12 (IQR 10, 15) years. Inclusion criteria were the diagnosis of displaced tibial shaft or dia-metaphyseal distal tibial fractures in patients of both genders, between 3 and 17 years of age treated with ESIN, followed up for a minimum of three months. Exclusion criteria were patients outside the predetermined age range, patients with conservative treatment of fractures or treated operatively with other methods (expert tibial nail, external fixation, plating), patients with proximal tibial fractures, patients with epiphyseal injuries, patients with closed physis or body weight > 70 kg, patients with follow-up shorter than three months, and patients with incomplete data. For the purpose of this study, the patients were subdivided in two subgroups regarding the localization of the fracture. The patients from the first subgroup had a tibial shaft fracture, while the patients from the second group had a distal tibial fracture. The outcomes of treatment were compared between the groups.

The Institutional Review Board (IRB) of our hospital approved the study (IRB reference, 500-03-/22-01/16, date of approval 3 March 2022). 

### 2.2. Outcomes of the Study and Hypothesis

The primary outcome of the study was the success rate of the ESIN method in the treatment of lower leg fractures, which is presented as a number of postoperative complications. Postoperative complications included wound complications, bleeding, fragment angulation, and refracture. Secondary outcomes were the time of bone healing, length of hospital stay, and associated injuries.

**Hypothesis** **(H1).** The ESIN is an effective method of treating lower leg fractures in children with excellent healing results and a low complication rate.

### 2.3. Study Design

This study was designed as a retrospective cross-sectional cohort study. According to the structure, this study was categorized as qualitative research, while according to the intervention and data processing, it was of a descriptive type. The source of data were electronic case records of the patients. All patients underwent emergency surgery, using the ESIN method, due to lower leg fractures. The age, gender, type of fracture, indication for surgery, fracture mechanism, lateralization, associated injuries, healing time, operative time, complications of treatment, and time to nail removal were analyzed for each patient. 

### 2.4. Radiographic Assessments and Indications for Surgery

All children underwent full-length anteroposterior (AP) and lateral (LL) radiographs of the lower leg. Displacement was assessed on mentioned radiographs.

The indications for surgery were open fractures, polytrauma, loss of reduction after conservative treatment, compartment syndrome, or initially severely displaced and unstable fractures (displacement for more than two-thirds of the diameter and/or angulation > 30° after manipulation). In regard to type of fracture, the most commonly severely displaced mild oblique, transverse, and spiral fractures were selected for surgery. Age limit was not strictly selected but the patients with closed physis and body weight > 70 kg were treated using an expert tibial nail.

### 2.5. Surgical Procedure

The surgery was performed under general anesthesia in the supine position. Titanium intramedullary nails (TEN; Synthes^®^ GmbH, Oberdorf, Switzerland) were used in all patients. The diameter and length of the nails were selected according to the bone length and child’s age (two nails must fill at least two-thirds of the medulla at the narrowest part of the bone). After preparation of the surgical field, the fracture site and proximal tibial epiphysis were marked under fluoroscopy. Two mini longitudinal incisions were made on the medial and lateral side at the level of the tibial metaphysis, proximal to the desired bony entrance. The starting point for nail insertion was located 1.5–2 cm distal to the bone epiphysis below the tibial tubercles. The cortical bone was shown by blunt dissection of soft tissues. Before the insertion, the nails were manually bent into a slight “C” shape that allowed fixation of the nail at three points. A drill was used to pierce the bone cortex which made it easier for the nail to enter the intramedullary canal. Both nails were then introduced into the intramedullary canal through the input incisions anteromedially and anterolaterally to the level of the fracture. Under fluoroscopic supervision, the fracture was reduced in both planes. After fluoroscopic confirmation, the first nail continued to advance toward the distal metaphysis of the tibia. If AP and LL X-rays confirmed that the distal position of the first intramedullary nail was correct, the second nail was inserted. Both titanium nails were pushed through the intramedullary canal towards the distal until their tips reached just above the distal tibial epiphysis, with special attention paid to not crossing the distal tibial physis, and at the end of the procedure, shortened at subcutaneous level. The surgical incisions were sutured using non-absorbable nylon sutures. After surgery, there was no casting in any of the age groups and physical therapy was started on the second or third postoperative day, depending on the patient’s condition and/or associated injuries.

### 2.6. Pain Management, Physical Therapy, and Follow-Up

All the patients were kept in hospital after the surgery. Most children with tibial fractures have the strongest pain within the first 48 h after the injury and use analgesia mostly for three days after injury. Our protocol includes fentanyl in a dose of 1.5 μg/kg immediately after surgery. After that period, Ibuprofen in a dose of 10 mg/kg and paracetamol in a dose of 15 mg/kg, individually or in combination, are the analgesics most commonly used, with no clear superiority.

**Figure 1:**
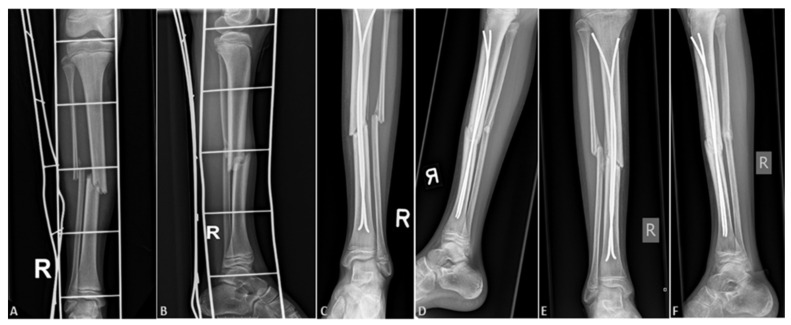
Displaced tibial shaft fracture in 11-year-old female patient: (**A**) preoperative AP radiograph; (**B**) preoperative LL radiograph; (**C**) AP radiograph one month after surgery; (**D**) LL radiograph one month after surgery; (**E**) AP radiograph three months after surgery; (**F**) LL radiograph three months after surgery.

**Figure 2:**
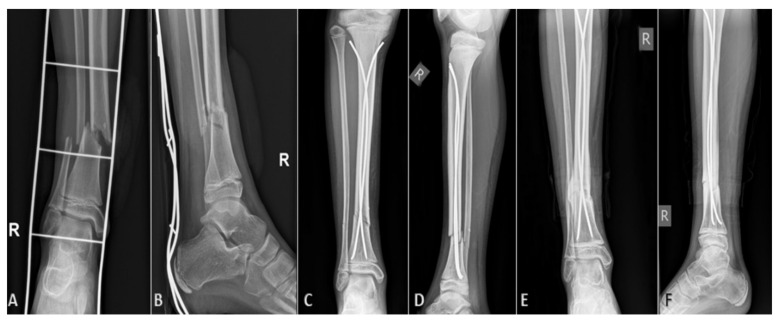
Displaced distal tibial fracture in 9-year-old male patient: (**A**) preoperative AP radiograph; (**B**) preoperative LL radiograph; (**C**) AP radiograph one month after surgery; (**D**) LL radiograph one month after surgery; (**E**) AP radiograph three months after surgery; (**F**) LL radiograph three months after surgery.

On the first postoperative day, strict rest is required, mostly due to pain control. From the second or third postoperative day (individually, depending on age and other factors, such as general condition of the patient or associated injuries), physical therapy and getting out of bed with crutches begins. After the patient learns to walk stably using crutches, if the other parameters are satisfactory, the patient is discharged to home care, continued with ambulatory physical therapy after discharge. For the first three or four weeks, the patient does not step on the operated leg, and then, after radiological verification of the fracture, begins gradual weight bearing. For the first few days, the bearing is approximately one-seventh of the body weight, after which the bearing gradually increases. On average, after 8 to 10 weeks of osteosynthesis, after radiologically verifying a good callus, a crutch-free gait begins. Each patient underwent an intraoperative X-ray after repositioning the bone fragments and placing of titanium elastic nails. Control X-rays were taken seven days after the procedure, and after one, three, and six months, or until healing of the bone was completed (Figure 1). Radiological evaluation was carried out using standard AP and LL radiographs at each visit to evaluate the consolidation of the fracture and identify complications such as secondary displacement, shortening, nail migration, delayed union, nonunion or malunion, and re-fracture (Figure 2). Nonunion was defined as the lack of appropriate healing within six months from index surgery. Malunion was defined as angular deformity of greater than 5–10° (depending on patient’s age) in the coronal or sagittal plane. Limb length inequality >1 cm was considered as limb shortening. All nails were removed under general anesthesia when the radiological healing was evident at the median of six months.

### 2.7. Statistical Analysis

The data were analyzed using Microsoft Excel for Windows Version 16.0 (Microsoft Corporation, Redmond, WA, USA), and Statistical Package for Social Sciences, version 19.0 (IBM SPSS Corp, Armonk, NY, USA) software programs. Distributions of quantitative data were described by medians and interquartile range (IQR), while categorical variables were expressed in absolute numbers and percentages. Differences in median values of quantitative variables between the examined groups were tested by the Mann–Whitney U-test. A comparison of different categories of variations was performed by the Chi-square test. In cases where the frequency rate of individual variants was low, Fisher’s exact test was used. All values of *p* < 0.05 were considered statistically significant.

## 3. Results

In the selected study period, which included 132 children operated on using ESIN for tibial fractures, there were 92 (69.7%) boys and 40 (30.3%) girls. The median age was 12 (IQR 10, 15) years. There were 100 (75.8%) closed and 32 (24.2%) open fractures. Median duration of hospital stay was 4.5 (IQR 2, 5) days. Most of the patients (*n* = 111 (84.1%)) had a fracture localized in the shaft of the tibia, while the other 21 (15.9%) had a fracture localized in the distal part of the bone (Table 1). A total of 32 complicated fractures were recorded. Using Gustilo - Anderson classification a total of 16 fractures (50%) were categorized as grade 1, 13 (40.6%) as a grade 2 and 3 (9.4%) as a grade 3. Most common mechanism of injury was traffic accident (31.8%) and the most common type of fracture was complicated fracture (24.2%) (Table 2).

Statistical comparison of data between patients who had open and closed fractures showed that children with open fractures were significantly older than children with closed fractures (13.5 years (IQR 10, 15) vs. 11 years (IQR 8.5, 14.5); *p* = 0.031). Furthermore, children with open fractures had a significantly longer hospital stay (7 days (IQR 5, 9) vs. 3 days (IQR 3, 6); *p* = 0.001), a higher rate of associated injuries (*n* = 14 (43.7%) vs. *n* = 23 (23%); *p* = 0.022), and a higher rate of postoperative complications (*n* = 7 (21.9%) vs. *n* = 8 (8%); *p* = 0.031). No statistically significant difference was found between the examined groups in relation to the sex of the patient (*p* = 0.308), duration of surgery (*p* = 0.301), and lateralization of the fracture (*p* = 0.758).

Associated injuries were reported in 37 (28%) children with tibial fractures. Their incidence and severity were directly related to the mechanism of injury. Most of the associated injuries have been reported in children with complicated tibial fractures who have been exposed to high kinetic energies. There was a total of 60 different associated injuries reported (Table 3).

An appropriate fragment position was achieved by closed reduction in 116 (87.9%) patients and 16 (12.1%) patients required open reduction due to repositioning difficulties or soft tissue interposition. No intraoperative complications were recorded. Postoperative complications were reported in 15 (11.4%) patients (Table 4). Most complications (*n* = 9; 60%) were minor complications, mostly related to the wound at the nail insertion site, which were managed conservatively. Six (4.5%) patients required reoperation due to angulation of the fragments or refracture. In distal fractures of the tibia, the rate of postoperative complications was slightly higher (14.3%) compared to the fractures localized in the tibial shaft (10.8%).

Complete radiographic healing was achieved in the majority of the patients at a median of 7 (IQR 6, 9) weeks. The implants were removed under general anesthesia after healing without any complications at the median time of 6 (IQR 5, 8) months. After removal of the intramedullary nails, all patients regained full limb function and all complications were successfully resolved. All patients were followed until the end of this study and the median follow-up time was 118.5 (IQR 74.5, 170) months.

## 4. Discussion

Pediatric fractures of the tibia can generally be managed by a non-operative approach [20,21,22]. However, this conservative type of treatment requires prolonged immobilization, careful follow-up, and complications such as secondary displacement, angulations, muscle atrophy, and refractures are not rare [23]. In younger children, up to four years of age, most of the lower leg fractures occur from falls or torsional forces, causing spiral and oblique tibial fractures. The fibula is usually intact in these fractures, preventing shortening but risking varus deformity. Older children usually suffer from indirect sporting injuries or direct injury from motor vehicle trauma, where both bones are usually involved. The cases with isolated tibial fracture usually are stable, not severely displaced or shortened, and may be usually treated conservatively with closed reduction and casting. Contrary to the above, the cases with both bones involved are usually displaced and require reduction and surgical treatment due to instability or shortening [3,4,13,22].

In recent years, the number of surgical procedures and indications for surgical treatment has significantly increased [8]. The ESIN method is currently a gold standard for the treatment of diaphyseal fractures in the pediatric population and adolescents [16]. Invention of the ESIN method gave an opportunity to children who sustained a fracture of a long bone that their time of hospitalization and immobilization can be significantly shorter. Moreover, this method became very popular because it is highly effective, complications are usually minor, and potential damage to the epiphyseal growth plate is minimized [21,22]. Intramedullary titanium nails provide stable and elastic fixation of the bone fragments, which allows controlled motion at the fracture site and provides quicker healing [24]. ESIN for the treatment of pediatric tibial fractures results in reliable healing for a majority of patients, but at the same time poses risks for angular deformities and delayed healing. Open fractures and compartment syndrome were associated with adverse radiographic outcomes [25].

The results obtained from the present study clearly showed that ESIN is a safe and effective method for treatment of tibial shaft and dia-metaphyseal distal tibial fractures with a low number of complications and relatively short length of hospital stay. Most of the complications were graded as minor (entry skin irritation, inflammation of the wound), while severe complications such as angulation of the fragments or refracture were rarely reported. Most of the authors recommend casting for a few weeks after surgery, probably due to fear of displacement of fragments or angulation [21,26,27,28]. In this study, we clearly showed that titanium intramedullary nailing may be safely performed without casting and one of the most important benefits of this method is early physiotherapy. Several previous reports on upper and lower extremities support this constancy [13,15,17,18,19].

Swindells and Rajan performed a systematic review of seven different retrospective studies regarding tibial ESIN with outcomes of 210 patients [29]. The authors of those studies described several indications for use of ESIN in pediatric patients, but most of them stated that the main indication was unstable fractures. The longest mean healing time in those studies was 20.7 weeks, reported by Srivastava et al. [21], and the shortest was 7 weeks, reported by Kubiak et al. [24]. Reported complication rates were similar, ranging from 12% to 35%. The most commonly reported complications were delayed union, malunion, nonunion, leg length discrepancy, and infections. All seven studies concluded that ESIN is an effective and safe method for treatment of unstable fractures of the tibial shaft in children and adolescents. They also concluded that most of the pediatric tibial fractures can be treated by a non-operative approach, but in cases where the surgery cannot be avoided, ESIN provides an acceptable and valuable option.

Griffet et al. reported that all 86 children included in their study were able to have unrestricted physical activity six months after the treatment with the ESIN method for tibial fractures [30]. They showed that the fixation of pediatric diaphyseal tibial fractures using ESIN is an effective method of treatment in pediatric patients and adolescents. 

Uludağ et al. published a study which included 20 children. The mean time of radiographic healing was 11 weeks and there were six (30%) instances of irritation and infections at the nail entry site [31]. Furthermore, they reported that three of six patients with an open fracture had infections of the wound. All their patients gained full range of motion of the ankle and knee joints. They concluded that intramedullary fixation with ESIN provides favorable outcomes in the treatment of unstable pediatric tibial shaft fractures that cannot be reduced with conservative treatment modalities.

Onta et al. reported that their 18 patients had a mean hospitalization time of 5.7 days and that all children achieved radiographic healing at the mean time of 13.3 weeks [32]. All of their patients had excellent final results and all of them had full range of movement at the knee joint. Four children had minor complications (nail protrusion and skin irritation), which were managed non-operatively. They concluded that ESIN had benefits of low blood loss compared to plating, and they also reported easier nursing care, early ambulation, and no complications of prolonged immobilization. 

Shen et al. published a study with 21 children that went under the surgery procedure with the ESIN method due to severely displaced dia-metaphyseal distal tibial fractures. They reported a mean hospitalization time of 3.9 days, and the mean time of the nail removal was 7.1 months [33]. A total of 19 patients achieved radiographic healing at the mean time of 9.6 weeks and two patients had delayed healing 10 months after the surgery. Their study showed good functional and radiological results in the pediatric population who had severely displaced DTDMJ (distal tibial diaphyseal metaphyseal junction) fractures that could not be casted. 

Kc et al. reported that in their study, which included 45 children treated with the ESIN method due to fracture of tibia, that they had a mean healing time of 11.17 weeks [34]. They recorded 20 postoperative complications which included 2 malunions, 4 delayed unions, 3 limb shortening, 2 limb lengthening, 6 nail prominences and skin irritations, 2 superficial infections on the nail entry site, and 1 refracture. However, none of their patients required secondary surgical intervention due to those complications. They concluded that ESIN is a simple, easy, reliable, and effective method for management of pediatric tibial fractures with shorter operative time, lesser blood loss, shorter length of hospital stay, and adequate time for bone healing.

Pennock et al. in their study compared 44 patients who underwent ESIN with 26 patients who received open reduction with internal fixation (ORIF) for tribal shaft fractures [28]. Patients that underwent ORIF had a longer mean surgical time and their mean casting time was seven weeks; minor complications were recorded in 10 (38%) patients and major ones were recorded in 3 (12%) patients. Patients that underwent ESIN had a shorter surgical time, and their mean casting time was 10.5 weeks; minor complications were recorded in 12 (27%) cases, while major complications were recorded in 8 (18%) cases. They concluded that patients treated with ORIF tend to heal and mobilize a few weeks faster, they have slightly more anatomic reductions at final healing, and they are less likely to require implant removal. Moreover, they pointed out that both ESIN and ORIF treatments contribute to a faster return to activities and that potential advantages of ORIF must be balanced with the potential increased risk of wound complications.

There is no exact consensus among the authors which age or height should be set as a limit for ESIN. Many authors used ESIN for treatment of pediatric and adolescent tibial fractures without any limits [13,22,33,35]. In previous studies, the rate of delayed healing was shown to vary by around 10% [13,22,33]. Gordon et al. in their study reported that patients with delayed union were older and they concluded the lack of stability may be the key factor behind the delayed healing [22]. Although there is a wide age (height) range in the majority of the studies, age of the patients ranges between 10 and 12 years of age [13,22,23,26,33,35,36]. A recent study showed that patients with tibial fractures who weigh 50 kg or less and with proximal tibial growth plates wide open can be treated with elastic stable intramedullary nailing, while more mature adolescents benefit from rigid intramedullary nailing as rigid nailing allows more precise fracture alignment without increased risk of growth disturbance [36]. Similar findings were observed in another recent study [37]. Hanf-Osetek et al. in their study compared children weighing less than 50 kg or more than 50 kg and found that the use of ESIN in displaced tibial shaft fractures in growing children weighing 50 kg or more is acceptable and safe [38]. A recent study performed by Thabet et al. compared adolescents treated for tibial shaft fractures using ESIN, interlocking nails, plates, and screws or external fixators and showed that open fractures had higher complication rates but no statistically significant differences in complication rates between the fixation methods was observed [39]. In general, the good results using the ESIN method may be obtained when the surgeon has a good knowledge of the method, respects and understands indications for surgery, and the main principles of the correction of the fracture and its stability [40].

In our study, of the sample of 132 children, the mean time of radiographic healing was seven weeks. We recorded complications in fifteen (11.36%) patients, which included five angulations of the fragments, four entry site irritations, two protrusions of the nails, two skin blisters, one pseudoaneurysm, and one refracture. Six (4.5%) of our patients needed a reoperation due to angulations of the fragment and refracture. The median of surgical time was 56 min. These results are similar to previous reports. Although most of the previously mentioned studies reported usage of a postoperative cast or a slab, our department policy is that after the surgical procedure with ESIN, no type of immobilization is needed. According to our positive results of this study, which included 132 patients, we proved that cast immobilization is not needed after the surgery procedure with the ESIN method due to tibia fracture. 

The results of this study must be interpreted within the context of several limitations. First, this was a single-center study, and the data were collected retrospectively. Furthermore, sample size was relatively small (although significantly higher than in most of the published reports). Next, there was a lack of a comparison group because a very low number of the patients were treated with other surgical techniques (such as plating, external fixation, or expert tibial nail). The majority of pediatric tibial fractures can be successfully treated conservatively by non-operative management and only the patients who fail conservative treatment (or are open/polytrauma/initially unable to reduce) are selected for surgery (most commonly ESIN). Accordingly, we were not able to design an adequate control group. Moreover, as this was a retrospective study, we could not find the data regarding functional outcome for the majority of the patients, especially for those operated on in earlier years. Prospective, multicenter studies with a larger sample size need to be conducted in the future before any definite conclusions in this regard should be drawn.

## 5. Conclusions

ESIN fulfills all criteria of minimally invasive bone surgery and is a highly effective treatment for pediatric tibial unstable fractures with a low rate of complications. Furthermore, the results of this study clearly showed that titanium intramedullary nailing may be safely performed without casting, and one of the most important benefits of this method is early physiotherapy.

## Figures and Tables

**Table 1:** Demographic data of the patients and clinical characteristics of fractures.

Patient Characteristics	All Fractures (*n* = 132)	Open Fractures (n = 32)	Closed Fractures (*n* = 100)	*p*	Distal Fractures (*n* = 21)	Diaphyseal Fractures (*n* = 111)	*p*
Age	12	13.5	11	0.031 *	12	12	0.499 *
Median; years (IQR)	(10, 15)	(10, 15)	(8.5, 14.5)		(10, 15)	(9, 15)	
Sex; *n* (%) Male	92 (69.7)	20 (62.5)	72 (72)	0.308 ^†^	15 (71.4)	77 (69.4)	0.850 ^†^
Female	40 (30.3)	12 (37.5)	28 (28)		6 (28.6)	34 (30.6)	
Lateralization; *n* (%)							
Left	67 (50.8)	17 (53.1)	50 (50)	0.758 ^†^	11 (52.4)	56 (50.5)	0.871 ^†^
Right	65 (49.2)	15 (46.9)	50 (50)		10 (47.6)	55 (49.5)	
Time of healing	7	10	7	0.015 *	6.5	7.5	0.374 *
Median; weeks (IQR)	(6, 9)	(10, 12)	(6, 9)		(6, 7)	(6, 9)	
Hospital stay	4.5	7	3	0.001 *	4.5	3.5	0.354 *
Median; days (IQR)	(2, 5)	(5, 9)	(3, 6)		(3, 6)	(3, 5)	
Duration of surgery Median; min (IQR)	54 (47, 66)	56 (49, 70)	53 (48, 64)	0.301 *	58 (46, 67)	56 (49, 59)	0.411 *
Associated injuries; *n* (%)	37 (28)	14 (43.7)	23 (23)	0.022 ^†^	3 (14.3)	34 (30.6)	0.185 ^‡^
Complications; *n* (%)	15 (11.4)	7 (21.9)	8 (8)	0.031 ^†^	3 (14.3)	12 (10.8)	0.645 ^‡^

* Mann–Whitney U test; ^†^ Chi-square test; ^‡^ Fisher’s exact test; IQR—interquartile range.

**Table 2:** Distribution of the patients according to fracture type and mechanism of injury.

Mechanism of Injury	Distal Fracture (*n* = 17)	Oblique Fracture (*n* = 25)	Comminuted Fracture (*n* = 14)	Complicated Fracture (*n* = 32)	Transverse Fracture (*n* = 18)	Spiral Fracture (*n* = 26)
Fall from height (*n* = 9)	1	2	1	2	1	2
Fall in same level (*n* = 29)	5	5	3	4	4	8
Road traffic accident (*n*= 42)	5	9	1	21	1	5
Sport (*n* = 21)	1	5	1	2	6	6
Bicycle riding (*n* = 10)	2	2	2	0	3	1
Motorbike (*n* = 18)	1	2	5	3	3	4
Electric scooter (*n* = 3)	2	0	1	0	0	0

**Table 3:** Associated injuries of children who underwent ESIN due to tibial fracture.

Associated Injuries	*n*	%
Excoriations and wounds	24	40
Epiphysiolysis and fractures of long bones	13	21.7
Soft tissue hematomas	9	15
Parenchymal organ injuries	4	6.6
Fractures of short bones	3	5
Teeth injuries	2	3.3
Nerve injuries	2	3.3
Serial rib fracture and pneumothorax	1	1.7
Fracture of pelvis	1	1.7
Subarachnoid hemorrhage	1	1.7
Total	60	100

**Table 4:** Postoperative complications.

Complication	*n*	%
Angulation of the fragments	5	33.3
Entry skin irritations	4	26.7
Protrusions of the nails	2	13.3
Blisters	2	13.3
Pseudoaneurysm	1	6.7
Refracture	1	6.7
Total	15	100.0

## Data Availability

The data presented in this study are available upon request of the respective author.
